# Multisampling Strategies for Determining Contaminants of Emerging Concern (CECs) in the Marine Environment

**DOI:** 10.3390/jox15050149

**Published:** 2025-09-15

**Authors:** Enrique J. Díaz-Montaña, Sofía Domínguez-Gil

**Affiliations:** 1 Department of Analytical Chemistry, Faculty of Pharmacy, University of Seville, 41012 Seville, Spain; 2 School of Chemical Science, Dulin City University, D09 YT18 Dublin, Ireland; sofiadominguezgil@gmail.com

**Keywords:** sampling evaluation, grab water, passive sampling, biofilm mesocosm, contaminants of emerging concern, marine environment

## Abstract

The determination of contaminants of emerging concern (CECs) in the marine environment is performed through many different sampling approaches. Therefore, the main objective of this study was to compare different existing sampling strategies: biofilm mesocosms, considering micro- and macrofouling; passive sampling; and grab marine water. The sampling of grab water was performed considering spatial and time-line variations. The spatial analysis of CECs showed that three sun agents and caffeine represent the biggest proportion of CECs in the Malaga Mediterranean coastal area, ranging from 0.391 to 0.495 ng/L. The time-line analysis did not show any upward or downward trend in CEC concentration. The mesocosm study comprised a separate evaluation of micro- and macrofouling that showed similar profiles, in which the sun agents presented the highest concentrations. While certain compounds were detected at comparable levels in both fouling types, such as clotrimazole around 0.001 ng/L, others exhibited significant differences in concentration, like caffeine. The passive sampling was also performed, obtaining similar results to those observed in the biofilm mesocosm macrofouling. Finally, all the obtained results from the different samplings were statistically compared, showing that passive sampling presented greater similarities with macrofouling and that there are significant differences between the sampling approach employed.

## 1. Introduction

Coastal environments are characterized by the dynamic interface between land and sea and harbour rich biodiversity and essential ecosystem services vital for human well-being. However, these ecosystems face pressure from anthropogenic activities, including urbanization, industrialization, and pollution, which threaten ecological integrity and resilience [1]. Among all the pollutants reaching the coastal and marine environments, new chemicals known as contaminants of emerging concern (CECs) stand out as a threat to human life and wildlife. These potential risks to aquatic ecosystems and human health are due to their persistence, bioaccumulation, and adverse effects. These compounds originate from various sources including pharmaceuticals, personal care products, and chemistry industries, and have garnered increasing attention for their ubiquity and potential ecological impacts [2]. The presence of CECs in coastal waters emphasizes the need for comprehensive monitoring programs to assess their occurrence, fate, and ecological risks [3].

The monitoring of CECs strongly depends on the matrix to be analysed, i.e., water (surface water, ground water, sea, open sea, wastewater, etc.), soil, or air. The common practice to monitor CECs in water is grab sampling, which is bottling water from the location of interest, or passive sampling [4]. Passive sampling (PS) techniques have gained interest in recent years because they allow the quantification of compounds in water, offering more efficient storage and deployment without compromising sensitivity [5,6]. There are many commercially available receiving phases, and modifications of the existing ones are increasing their utility, enhancing the use of PS for water sampling purposes [4]. However, PS also has disadvantages, such as the need of calibration prior to its placement, limited information on the capture efficiency of many sorbent materials, and the inability to collect biological analytes [7]. Furthermore, biota has emerged as a new monitoring strategy for complex environments, like sea/coastal waters, due to the inconvenience presented by PS. A biofilm mesocosm is a combination of multiple organisms (biota) and microorganisms, and it offers advantages as a sample medium for CEC monitoring. A biofilm is a complex assemblage of microorganisms embedded within a matrix of extracellular polymeric substances, forming resilient structures known for their diverse ecological roles [8]. These structures, pervasive in aquatic environments, act as dynamic reservoirs of microbial diversity and metabolic activity, influencing nutrient cycling, pollutant degradation, and biofilm formation processes [9].

Therefore, the objective of this study was to compare different sampling approaches: grab water, biofilm mesocosms (micro- and macrofouling organisms), and passive sampling. Due to the mobilization of contaminants in transitional and coastal areas, the grab sample was taken considering two perspectives: (i) to conduct a thorough examination of multiple sites along the coast of Malaga and (ii) to evaluate pollutant levels over an extended duration of five weeks. By leveraging the biofilm mesocosm’s capacity to retain and concentrate contaminants, this study aims to enhance the sensitivity and specificity of environmental monitoring efforts, providing data for assessing the occurrence of CECs and the impact for health and resilience of Malaga’s coastal ecosystem.

## 2. Materials and Methods

### 2.1. Chemicals

The chemical compounds identified by high-performance liquid chromatography (HPLC; Agilent 1260 series Infinity binary pump (Santa Clara, CA, USA)) coupled to a triple quadrupole mass spectrometer (MS/MS; Agilent 6470 (Santa Clara, CA, USA)) were confirmed using analytical standards (MS degree) purchased from Merck (Darmstadt, Germany) (Table 1). The quantification was performed employing a calibration curve with eighteen points, from 0.01 to 100 ng/L, for each compound. All the analysis, as well as the calibration curves, were performed nine times (in nonuplicate).

### 2.2. Monitoring Sites

**Figure 1:**
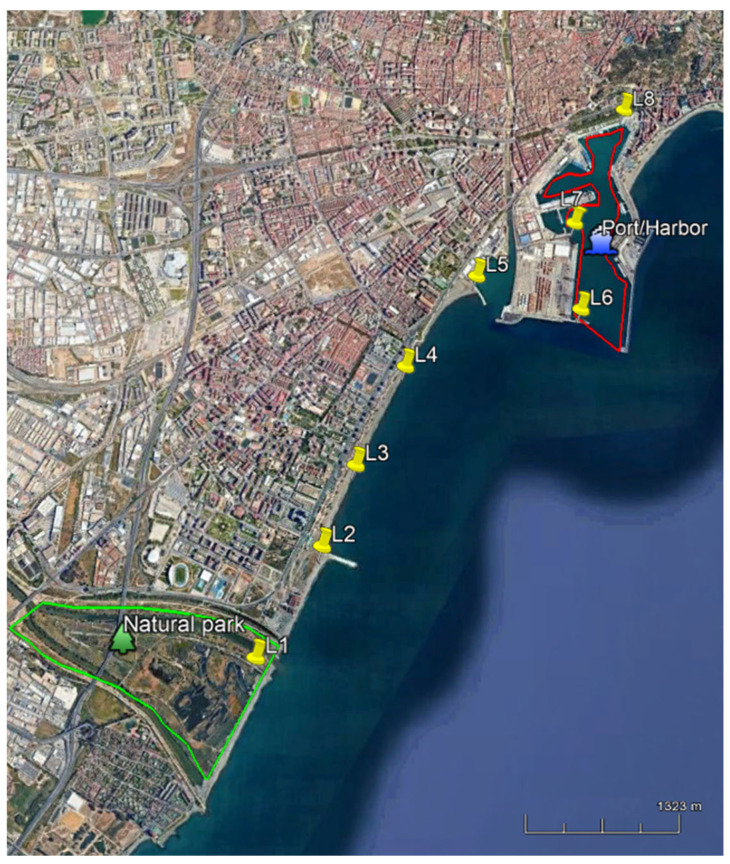
Sampling locations. Green line delimitates the green areas, whereas the red line delimitates the harbour/port area.

The spatial analysis of Malaga (Spain) coast was performed, as mentioned, in order to obtain information from the Mediterranean Sea about the pollutants ending up in it. Table 2 lists the selected sampling locations with the assigned code, the exact coordinates (longitude and latitude), and the main activity performed there. Figure 1 (included in Table 1) shows the sampling points, and the spatial analysis was conducted using Google Earth Pro software v. 7.3.6.10201.

The concentration variability along different weeks (time-line analysis) was performed in L5 (36°70′24.7″ N 4°42′60.3″ W), at the end of Guadalmedina River (transitional water), where swimming and navigation activities are performed close to the Malaga Port. This site was chosen because it is a tidal, highly dynamic ecosystem, with anthropogenic pressure caused by shipping activities or tourism, as well as the influence of the surface water from the river.

### 2.3. Sampling

Three different types of sampling have been performed, i.e., grab water sampling, biofilm mesocosm sampling, and passive sampling (Figure S1). The grab water sampling has been performed considering time-line analysis and spatial analysis, to improve the understanding of the location of interest. Figure S1 schematizes the different sampling types and deployment and retrieval periods.

#### 2.3.1. Grab Water Sampling (Spatial and Time-Line Analyses)

The time-line analysis samples were taken during five consecutive weeks starting in March 2025. However, the spatial analysis samples were taken in April 2025, coinciding with the last week of the time-line analysis.

Both spatial and time-line analysis sampling were performed using the “grab water approach”, using glass bottles, which were previously rinsed with methanol and double-distilled water, taking 1 L of seawater each time. Water samples were taken at 1 m from the surface and 10 m from the beach side. Samples were stored at −18 °C until extraction.

#### 2.3.2. Biofilm Mesocosm Sampling

The biofilm mesocosm cage holder was a modified lobster cage. It was soaked overnight (10 h) in DCN 90 surfactant detergent (Merck, Germany). Afterwards, it was washed thoroughly with ultrapure water, rinsed with methanol in an ultrasonic bath for 10 min, and, finally, air dried. Once the biofilm cage was cleaned, it was deployed in April 2024 at L5 using a rope at a depth of 1 m from the sea surface and was collected after twelve months, allowing the development of biofilm mesocosms, microorganisms, and macroorganisms.

#### 2.3.3. Passive Sampling

The passive sampling deployment was also conducted in March 2025 and at the L5 location site at 10 m from the biofilm holder to avoid possible bias; thus, the comparison between the different sampling procedures could be made. It was submerged for one month, leaving 1 m between the sampler and the water surface. As the target compound were pharmaceuticals, sun agents, and pesticides, hydrophilic–lipophilic balance (HLB) disks (Affinisep, France) were used. Furthermore, the lobster cages, i.e., the chemcatchers (PTFE bodies; Affinisep, France), were soaked overnight (10 h) in DCN 90 surfactant detergent (Merck, Germany). Afterwards, they were washed thoroughly in ultrapure water, rinsed with methanol in an ultrasonic bath for 10 min, and, finally, air dried. The HLB disks were conditioned as follows: firstly, soaking them overnight (10 h) in methanol; secondly, washing with 50 mL of methanol under gentle vacuum; thirdly, washing with 100 mL of distilled water under gentle vacuum; and finally, storing them in water until the deployment. Once the disks were prepared, they were covered by a polyethersulfone (PES) membrane (Affinisep, France), which was previously rinsed with methanol and water for 30 min each and stored in water until deployment, and then placed in a clean and dry chemcatcher (Affinisep, Le Houlme, France).

### 2.4. Sample Treatment

Each sample (biofilm mesocosm, PS, and grab water) underwent different initial preparatory procedures for the extraction of the target analytes. The microorganism biofilms, as well as the macroorganisms, were removed from the glass slides using a cell scraper to transfer them into large glass jars and were separated out based on the type of organism collected. Firstly, biofouling organisms were divided into two categories for collection: microfouling organisms (i.e., microalgae and slime) and macrofouling organisms (i.e., barnacles, tubeworms, red and green algae). Secondly, both types of fouling were dried in an oven (Moder Orbital Incubator S1500 Stuart, VWR, Avantor, Barcelona, Spain) at 30 °C until the weight remains constant. A 2 g quantity of (micro or macro) mesocosm was weighed in a glass vial, and 50 mL of mass spectrum (MS)-grade methanol (Honeywell, NC, USA) was added to each sample. Afterwards, the samples were sonicated for 20 min, filtered through a 0.45 µm nylon filter disk (Merck, Germany) into a falcon tube, and centrifuged (Model 5804, Eppendorf, Hamburg, Germany) for 20 min at 4000 rpm. Finally, the supernatant was transferred to a 100 mL brown bottles and spiked with 25 µL of 100 ng/L internal standard mix.

In the case of PS, the sampler surfaces were wiped with paper tissues and rinsed with seawater. The wrapped devices were transported in aluminium foil and placed in cool boxes. Then, the device (chemcatcher) was dissembled, and the PES membranes were discarded. The HLB disks were dried at room temperature on solvent rinsed foil. If the HLB disks need to be stored, once dried, they were kept at −4 °C. Before the extraction, the disks were left at room temperature for 2 h. The extraction of the HLB disks was carried out by placing them in a 100 mL clean beaker with 40 mL of LC-MS/MS-grade methanol and shaking them for 1 h. Then, the membranes were placed in a filtration system, and the methanol employed before was passed through them. The extract was collected in a 50 mL falcon tube and evaporated with nitrogen (N2) to dryness. Finally, it was reconstituted in 1 mL of methanol/water (20:80) and spiked with 25 µL of 100 ng/L internal standard mix.

The extracts from the mesocosm and the water samples were subjected to the same preconcentration step, employing OASIS HLB 500 mg solid-phase extraction (SPE) cartridges (Affisinep, Le Houlme, France). The cartridges were conditioned with 4 mL of methanol, followed by 4 mL of ultrapure water (adjusted with glacial acetic acid to pH 3). The samples (mesocosm supernatant and water) were loaded into the cartridge at a flow rate of 2 mL/min. The cartridges were rinsed with 4 mL of ultrapure water and were dried with vacuum for 30 min. Analytes were eluted with 5 mL of acetonitrile (ACN) (Honeywell, NC, USA) into amber vials (22 mL). Eluates were placed under a N_2_ current until dryness. Samples were then reconstituted in 1 mL methanol/water (20:80), vortexed, and left to stand in 22 mL vials for 5 min. The reconstituted sample was syringe-filtered with a 0.2 µm nylon filter (Merck, Germany) and transferred to an LC-MS/MS 2 mL vial for storage at 4 °C until analysis.

### 2.5. Instrumental Analysis (LC-MS/MS)

All extracts were analysed by high-performance liquid chromatography (HPLC; Agilent 1260 series Infinity binary pump) coupled to a triple quadrupole mass spectrometer (MS/MS; Agilent 6470), equipped with an electrospray ionization source. A 10 µL volume of the extracts was injected into an Agilent Zorbax Eclipse Plus C18 column (3.0 × 50 mm; 1.8 mm), with a guard column of the same materials. Mobile phases for pharmaceutical (Table 1) analysis were as follows: (A): 0.1% formic acid/5 mM ammonium formate (pH 3.2) in water and (B): 0.1% formic acid in acetonitrile; for pesticides and sun agents (Table 2), the mobile phases employed were as follows: (A) 0.1% formic acid/5 mM ammonium formate (pH 3.2) in water and (B): 0.1% of formic acid in methanol; in both cases, the flow rate (0.4 mL/min) and gradient were the same. The gradient elution was as follows: 0–1.5 min, 90–45% A; 1.5–7.0 min, 45–10% A; 7.0–8.5 min, 10–0% A; 8.5–10 min, 0% A; 10–11 min, returns to initial conditions; 11–12 min, equilibration of the column. Column temperature was set at 35 °C. Compound-dependent mass spectrometric parameters (fragmentor, collision energy) as well as compound-selected reaction monitoring transitions were optimized by the direct infusion of individual standard solution of each analyte at 10 ng/L using the Agilent Compound Optimizer software v. 12.1. The ionization mode employed for pharmaceuticals (Table 1) was positive (ESI+) and for pesticides and sun agents (Table 1) negative (ESI-). Source-dependent parameters were determined by flow injection analysis: gas temperature: 230 °C; gas flow: 4 L/min; nebulizer: 15 psi; source temperature, 375 °C; ion spray voltage, 2500 V; and sheath gas flow: 12 L/min. Instrument control data acquisition and data analysis were carried out using the Agilent software (MassHunter Workstation).

The analysis of each sample was performed in nonuplicate. The chemical identification performed by liquid chromatography coupled with a mass spectrum triple quadrupole (LC-MS/MS) was confirmed by the analysis of analytical standards (MS-grade) (Table 1) (Merck, 185 Darmstadt, Germany) and the monitored transitions.

### 2.6. Quality Assurance and Quality Control

All materials were washed with ultrapure water and rinsed with methanol three times; additionally, the glass materials were heated until dryness and rinsed with methanol again. Initially, three water blanks were analysed in nonuplicate to assure the absence of analytes of interest. Additionally, for each batch of ten samples, two water blanks were analysed in nonuplicate. Furthermore, all standards were randomly measured in a concentration range between 0.01 and 100 ng/L during the analytical run assuring the correct response of the instrument. All the blanks showed concentration below the method detection limit (MLD) for the different compounds.

Additionally, the analytical method was validated to ensure reliability and performance. The key validation parameters evaluated were linearity, matrix effects, selectivity, sensitivity, accuracy, and precision. As mentioned, the calibration curves were prepared between 0.01 and 100 ng/L. The linearity was considered acceptable when the correlation coefficient (r^2^) was greater than 0.99. The sensitivity was determined by the limit of detection (LOD) and limit of quantification (LOQ). The blank signal was multiplied by 3 to determine the limit of detection (LOD = S_blank_ × 3), and by 10 to determine the limit of quantification (LOQ = S_blank_ × 10). Accuracy and precision were assessed considering two concentrations (1 and 10 ng/L) in nonuplicate, being evaluated as recovery experiment-fortifying blanks. Acceptable values of recovery are considered between 70 and 120%, whereas precision (expressed as relative standard deviation (RSD)) has to remain below 20%. Matrix effects (MEs), expressed as percentage, were calculated as the ratio of the analytical signal obtained in a blank matrix to the signal obtained in the solvent. Lastly, selectivity was performed to ensure no interference at the target retention times, by comparing chromatograms of the blank and spiked samples.

### 2.7. Statistical Treatment

The data obtained from the Agilent software were exported to Excel 365 (Microsoft Corp., Redmond, WA, USA) to obtain graphs and basic statistics (mean, standard deviation, minimum and maximum concentrations). Once the basic statistics were performed and the homogeneity of variance was confirmed, the obtained information was used to perform an ANOVA for selecting the more important variables (compounds). Once the variables were selected, a principal component analysis (PCA) was performed. This multivariate analysis was performed using the Statistica software V8 (Statsoft; Tulsa, OK, USA).

## 3. Results and Discussion

Three different sampling approaches were considered (grab, mesocosm biofilms, and passive sampling). The grab water sampling was performed considering spatial and time-line distribution. Eight sampling locations along the Malaga Mediterranean coast, including beaches and harbours, were analysed to determine the occurrence of contaminants in the coastal marine area. Time-line analysis results were obtained to study the CEC variation over five consecutive weeks in the context of a busy dynamic coastal area. The twelve months deploying marine mesocosms provides a unique method to address potential differences between micro- and macrofouling uptake. Passive sampling was also employed to determine the occurrence of CECs in the Mediterranean coast. Lastly, the results obtained for the different sampling approaches were statistically compared to detect significant differences between them.

### 3.1. Method Validation

Before the sample analysis, the analytical method employed was validated (Table 3). The linearity was excellent for all the determined compounds (Table 1) with correlation coefficients ranging from 0.9912 (erythromycin) to 0.9994 (caffeine). The limit of detection (LOD) range was between 0.1 and 1.1 × 10^−^^3^ ng/L, whereas the limit of quantification (LOQ) range was between 0.2 and 3.3 × 10^−^^3^ ng/L. As mentioned, accuracy and precision were determined by recovery experiments at the fortification levels. The recovery rates were between 82.7% (carbamazepine) and 113.4% (prochloraz) at the lower spiking levels, while they ranged between 85.1% (gabapentin) and 111.9% (trimethoprim) at the higher spiking levels. The relative standard deviations (RSDs) were between 0.9% (o-desvenlafaxine) and 8.6% (avobenzone) at the lower spiking level and between 1.1% (ofloxacin) and 8.5% (oxybenzene) at the higher spiking level. Therefore, the acceptable criteria were satisfied for both accuracy (mean recovery within 70–120%) and precision (RSD below 20%). All the determined contaminants (Table 1) showed low-to-moderate matrix effects (MEs) ranging between −43.5% (carbamazepine) and 51.2% (bifenthrin).

### 3.2. Spatial Occurrence of Contaminants of Emerging Concern

**Figure 2:**
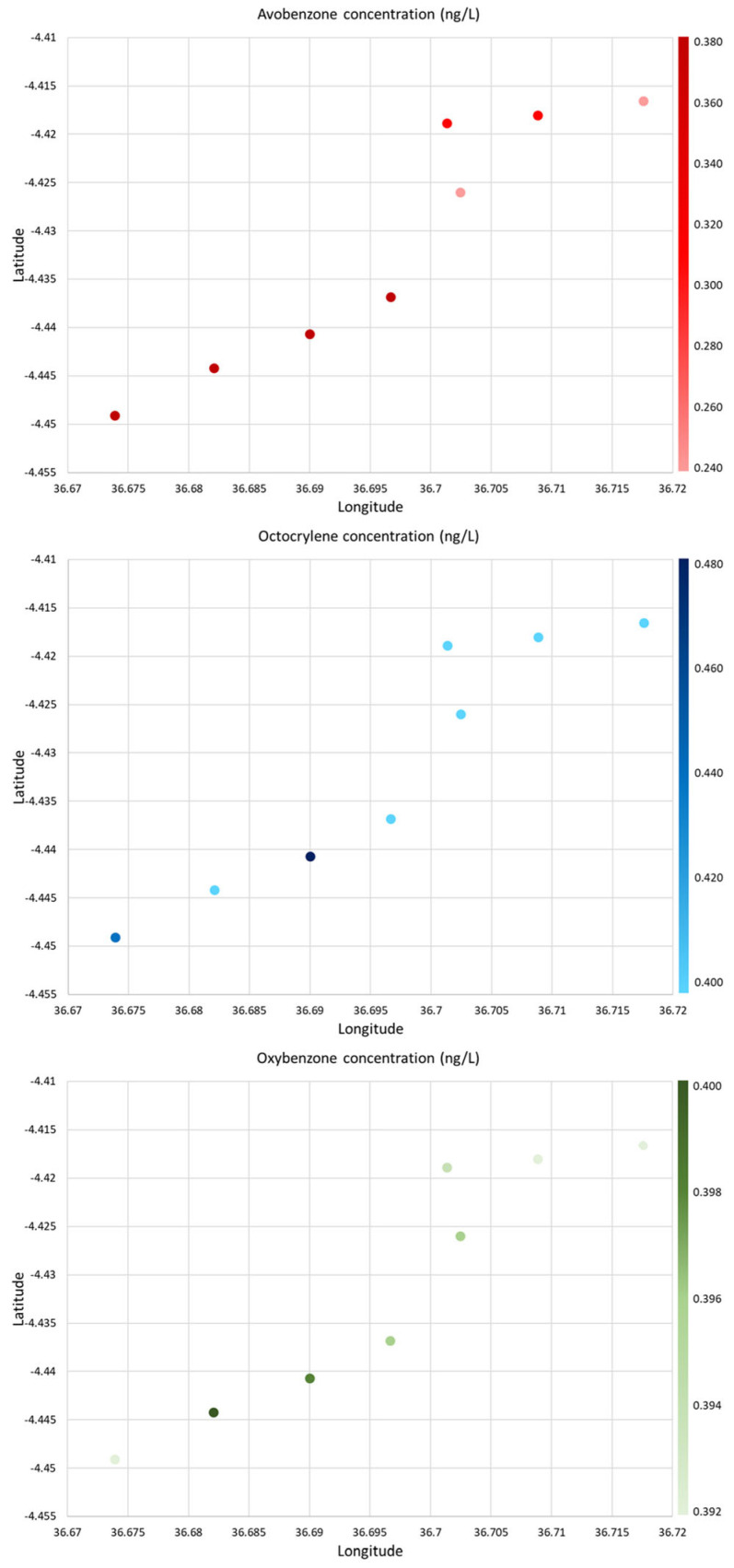
Concentrations (ng/L) of the sun agents in the different sampling points.

The twenty pollutants under study were determined in the eight sampling seawater sites, including L1 that is a protected natural park. The compounds that presented the highest concentration were the sun agents, followed by the pharmaceuticals and lastly the pesticides. The three sun agents quantified were avobenzone, octocrylene, and oxybenzone, with octocrylene being the most concentrated one in all the sampling sites. This compound is highly used due to its capacity of filtering UV radiation (UVF) and its capability of preventing erythema and, subsequently, the risk of melanoma due to sun exposure; but it presents an environmental problem due to its lipophilic character (log Kow = 6.88) that makes it resistant to degradation and stable in marine ecosystem [10]. The sun agents were more distributed in beach areas (L1–L4) than in harbours (L6–L8), which is logical given that harbours, unlike beaches, are primarily used for boating rather than swimming activities. The locations that presented the highest level of sun agents were the beach areas (Figure 2), especially L2 and L3 beaches presenting 0.495 ± 0.008 and 0.419 ± 0.009 ng/L of octocrylene, respectively. These results are in concordance with those of Fenni et al. [11] that concluded the necessity of UVF environmental monitoring due to the high usage and the ecotoxicity shown. However, as shown in Figure 2, the distribution was not homogeneous, having significant differences (*p* < 0.05) between the different sampling sites. As mentioned, the high concentrations of avobenzone and octocrylene in the protected natural park (L1) are astonishing. This demonstrates that the remobilization of these compounds into the marine environment threatens wildlife.

As the concentrations of sun agents were higher in the beach areas, the places that presented the highest concentrations of pharmaceuticals were those near the port (L6, L7, and L8), possibly due to the large number of cruises that directly dump their wastewater into the sea. As it can be seen in Table 4, the pharmaceuticals that showed the highest concentrations were caffeine and gabapentin. Additionally, the site L5 also presented a high level of caffeine, reaching a concentration of 0.391 ± 0.007 ng/L, and of gabapentin, i.e., 0.314 ± 0.006 ng/L, possibly due to the contamination coming from and near the Guadalmedina River, where sewage pipe systems directly dump waste into the sea. However, these concentrations are still far from the high caffeine concentration found on the Black Sea coast (13,575 ng/L) [12]. This difference is due to the sampling site, the sampling period (not tourist season—April—or tourist season—August), and the proximity to more or less densely populated areas. In contrast, the compounds that presented the lowest concentration were erythromycin and trimethoprim and reached 0.028 ± 0.004 and 0.081 ± 0.006 ng/L, respectively. These two drugs are antibiotics used for treating the infection of respiratory and urinary tracts, and their low concentration can be due to their fast deterioration rate or their relatively low consumption, compared to other antibiotics such as amoxicillin, in Spain. Additionally, the relative low concentration of these compounds can be due to the degradation of these chemicals in marine water as well as the inherent difficulty of marine water matrix, in concordance with Boti et al. [13].

The detection of the seven pesticides (Table 5) across the different sampling sites highlights a clear One Health concern. This framework recognizes that human health, animal health, and environmental integrity are interconnected. Some of these compounds, such as clotrimazole, fluconazole, and miconazole, are antifungals used in human medicine, while others, like bifenthrin or penconazole, are applied in agriculture or for pest control in grains and vegetables. Their simultaneous presence in coastal waters suggests multiple sources of contamination—both medical and agricultural—which converge in shared environments. The detection of bifenthrin at the highest concentrations, especially in L5 and consistently across all sampled beaches, together with the probable remobilization of contaminants via the Guadalmedina River as it passes through farming areas, indicates a transport pathway that integrates urban, rural, and aquatic ecosystems. Supporting this observation, studies on urban coastal and wetland sediments have shown that bifenthrin is frequently the most abundant pesticide, with concentrations capable of exceeding lethal thresholds for sensitive benthic organisms [14]. The environmental persistence of these substances, along with their endocrine-disrupting potential and capacity to act through diverse biological mechanisms, underscores the need for integrated management strategies that protect ecosystem health, safeguard wildlife, and prevent potential risks to human populations.

Therefore, these results underscore the necessity for a control of port areas and the direct dumping of waste, as well as the monitoring of surface water crossing highly populated and polluted areas.

### 3.3. Time-Line Analysis of CECs

The five-week analysis was performed at the L5 sampling site. As seen in the spatial analysis, it is a significant point due to its proximity to the Guadalmedina River, which has remobilized contaminants from land sources, and port and swimming areas, making this point a highly dynamic environmental area crucial for CEC monitoring.

**Figure 3:**
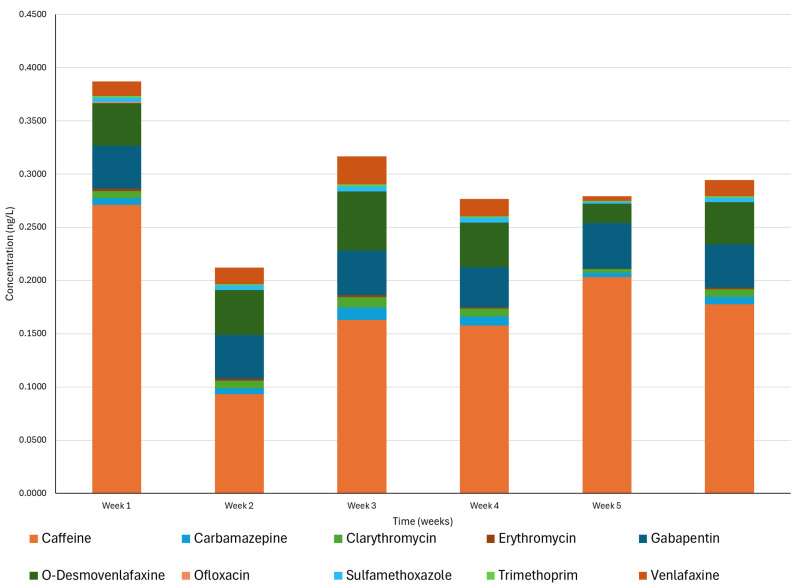
Predominance of individual and total pharmaceuticals (ng/L) in L5 sampling site during five consecutive weeks.

The sun agents were also compounds with the highest concentrations, but in this case, the compound that presented the highest concentration was avobenzone (Figure 3). This might be due to the difference in the sampling, while the previous analysis considered different locations in a short time, and this sampling was performed in five different weeks.

The pharmaceuticals showed the same trend as in the spatial analysis (Table S1), with caffeine the highest concentration, with a mean concentration of 0.178 ± 0.021 ng/L for the five weeks. The second most concentrated pharmaceutical was gabapentin (in the spatial analysis) and O-desvenlafaxine. These results highlight the limitation of grab sampling, not considering time variations. The ones with the lowest concentrations were erythromycin and trimethoprim, with 0.002 ng/L for both. Figure 3 shows a common behaviour among all the pharmaceutical compounds. Additionally, avobenzone may originate from water treatment processes, as studies have indicated that the use of showers, such as those found on beaches, can serve as a source of these contaminants [15].

Figure 3 also shows the cumulative sum across the various sampling weeks. As previously mentioned, the concentrations of these compounds ranged from nondetectable levels to 0.5 ng/L. No discernible trend was observed throughout the sampling period, which is a notable finding. This lack of trend suggests a consistent presence of marine pollutants of diverse origins contaminating the marine environment.

In the case of pesticides (Table S1), bifenthrin was detected at concentrations ranging from 0.030 ± 0.004 to 0.079 ± 0.007 ng/L, consistent with the spatial analysis for the same area. Sampling in different weeks enabled the detection of short-time-line variations and the remobilization within the marine environment. This was the case for pesticides, with four new compounds detected, i.e., diflufenican, famoxadone, mecoprop, and permethrin. As mentioned, the high diversity and abundance of pesticides near Malaga can be a consequence of the large number of green areas and of the river that starts in a mountainous/forest area, and in its 30 km length, it goes through several agricultural and farming areas, contributing to the presence of pesticides in Mediterranean Sea. In contrast, other authors have quantified up to thirty-seven pesticides in the Mediterranean French Coast [16]. Postigo et al. [17], in concordance with these results, concluded that the urban and industrial activities (like farming) are responsible for the occurrence of pesticides in the marine environment. On the other hand, the partial elimination of these pollutants in the wastewater treatment plants also contributed to the occurrence of these pollutants in the coastal and marine waters [18].

Additionally, a risk assessment was performed to evaluate the risk of the contaminants reaching the Malaga Mediterranean coastal area. The risk assessment (Table 6) was calculated by the risk quotient (RQ), which is the ratio of the measured environmental concentrations (MECs) between the predicted no-effect concentrations (PNECs): RQ = MEC/PNEC. Four risk levels were considered, i.e., RQ < 0.01—no risk (N.R.); 0.01 < RQ < 0.1—low risk (L.W.); 0.1 < RQ < 1—moderate risk (M.R.); and RQ > 1—high risk (H.R.). Table 4 shows the risk assessment of the compounds that have published and validated PNECs [19]; thus, not all the compounds are plotted. Four compounds showed low risk, i.e., caffeine, o-desvenlafaxine, venlafaxine, and azoxystrobin, even though caffeine was the most concentrated compound, but due to its high PNEC, it did not show high risk. However, the high concentration of caffeine it is a concern due to its relationship with oxidative stress, lipid peroxidation, and neurotoxicity [20,21]. Azoxystrobin was shown to be the most influent one depending on the measured weeks, presenting low risk in three of the five weeks. This needs to be considered when monitoring or with a longer time-line analysis, as certain compounds might show risk in certain periods, such as sun agents in the summer season. On the other hand, avobenzone and bifenthrin showed high risk due to their high concentrations and low PNECs (3.2 and 9.5 µg/L, respectively). The risk associated with avobenzone includes reproductive toxicity, developmental toxicity, neurotoxicity, and endocrine disrupting. Additionally, it has also been related to the development of nervous and retinal systems in zebrafish [22,23,24,25]. However, bifenthrin is associated with inducing hyperactivity and neurodevelopment issues (due to alterations in calcium oscillations) as toxic effects [26]. Therefore, these two compounds should be deeply studied to search for the sources of their production and considered for monitoring by the regulation institutions.

### 3.4. Biofilm Mesocosms as a New Contaminant Monitoring Tool

Biofilms are usually employed to study the microbial biofilms generated on marine plastics (the platisphere); however, macroorganisms can also develop on the surface of these devices or plastics [1]. Additionally, biofilms have been employed to remove dissolved organic compounds and the associated toxicity from produced water [9]. In this study, the mesocosm biofilms were studied considering the micro- and macrofouling uptake of thirteen target contaminants (seven were below the limit of detection (LOD)). Microfouling is the initial step in the growth of biofouling on hard substrata submerged in marine waters. Thus, for this fouling, bacteria and microalgae were analysed. However, macrofouling is the fouling caused by large organisms, such as macroalgae, oysters, mussels, clams, and barnacles. The thirteen CECs quantified included two sun agents (avobenzone and octocrylene), six pharmaceutical compounds (caffeine, carbamazepine, clarithromycin, gabapentin, O-desvenlafaxine, and trimethoprim), and five pesticides (azoxystrobin, bifenthrin, clotrimazole, penconazole, and prochloraz). Thus, not all the compounds detected in the spatial and time-line analyses were able to be detected in the mesocosm.

**Figure 4:**
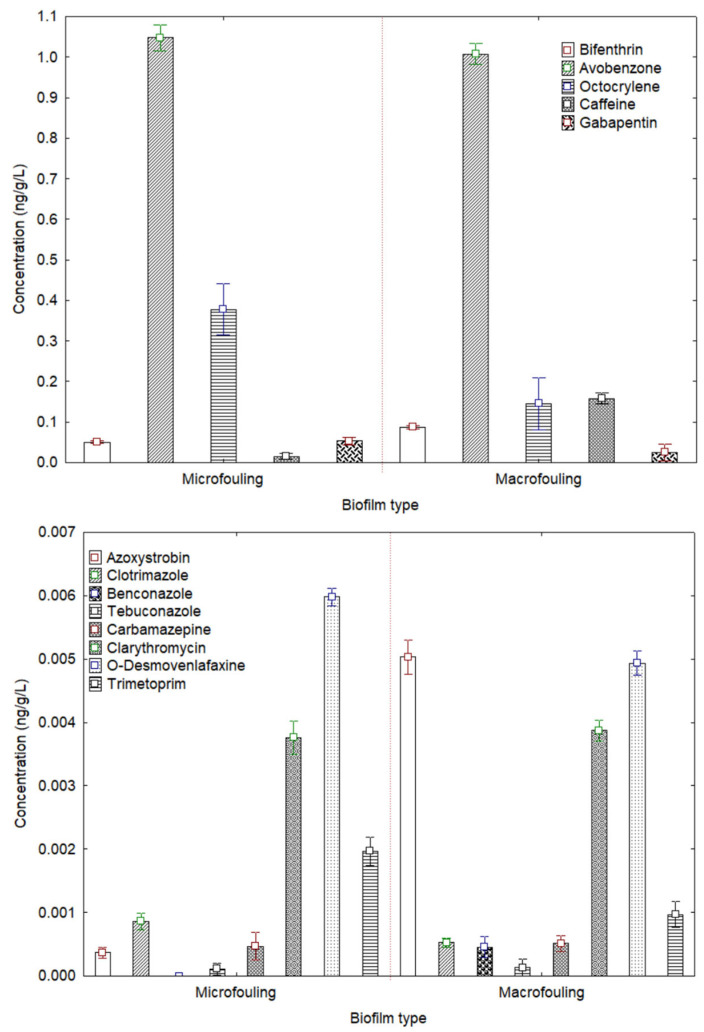
Box plot of high (**up**)- and low (**down**)-concentration compounds (ng/L) in both fouling of the mesocosm (separated by a red dotted line). Note: mean box: 25–75%.

In general, micro- and macrofouling showed the same profile (Figure 4), with sun agents being the compounds that presented the highest concentrations. Avobenzone, in the time-line analysis, was the most concentrated compound, with a significantly high concentration, having an average concentration for both fouling of 1.030 ± 0.030 ng/L. This highlights the differences that can be observed employing one type of sampling as well as the bioaccumulation of certain chemicals by organisms. Avobenzone and other UV filters, as mentioned, show toxicity and potential bioaccumulation in aquatic environments. Avobenzone has been quantified in significant concentrations in different aquatic environments due to the discharge from wastewater effluents, landfills, or recreational activities [27,28]. In contrast, octocrylene did present significant differences (*p* < 0.05) between both fouling (*p* < 0.05). For the mesocosm microfouling, the concentration of octocrylene was 0.381 ± 0.020 ng/L, whereas for macrofouling (mussels, barnacles, tunicates, and macroalgae), it was 0.150 ± 0.021 ng/L. These results are in agreement with the spatial and time-line analyses and have an advantage because they allow the quantification of the sun agents and give an output after a year of exposure (“year average”), whereas by other methodologies, octocrylene’s concentration varies depending on the day as observed for O-desvenlafaxine in the time-line analysis. The aquatic toxicity testing is complicated to perform because the octocrylene effect threshold is often above the water solubility limit [29]. In contrast to Pawloski et al. [30], certain bioaccumulation has been detected in the macroorganisms, but at it was mentioned in the study, there might be significant differences between organism bioaccumulation and the concentration quantified in water, possibly due to the season sampling period. However, biofilms consider one-year exposure, and water samples only consider the occurrence at the moment of sampling.

The pharmaceutical compounds exhibited consistent concentration levels across both fouling, except for caffeine. Caffeine showed significant (*p* < 0.05) disparity between microfouling and macrofouling. It was determined at 0.021 ± 0.010 ng/L in microfouling, whereas in macrofouling, the concentration surged tenfold to 0.164 ± 0.042 ng/L. This disparity aligns with the known bioaccumulative nature of caffeine in marine organisms, as reviewed in coastal systems where caffeine has been found in tissues of microalgae, bivalves, and fishes, producing different toxic effects [21]. As for the spatial and time-line analyses, the second most concentrated compound was gabapentin, confirming the previous data. In the case of erythromycin and trimethoprim, the first one was not detected, highlighting the influence on the sampling type and period, whereas trimethoprim was the least concentrated pharmaceutical compound. Trimethoprim was the least concentrated pharmaceutical, yet environmental risk assessments in European waters highlight its widespread presence, arguing for integrative sampling approaches that better reflect long-term exposure [31]. These results shed light on the importance of having a sampling method that could provide information for a long period, not only of the moment of sampling.

Conversely, the detection of pesticides yielded a limited number, with only five compounds identified. The pesticides, in the other sampling approaches, were the compounds that presented the lowest concentration. Among them, bifenthrin was the predominant one, exhibiting a concentration level that was a striking 100 times higher than that of the other pesticides detected, with an average between fouling of 0.074 ± 0.022 ng/L. The remarkably high concentration of bifenthrin in comparison to other pesticides can be due to its remobilization by the Guadalmedina River, sewage pipes, and it relation with residential and industrial areas [32].

**Figure 5:**
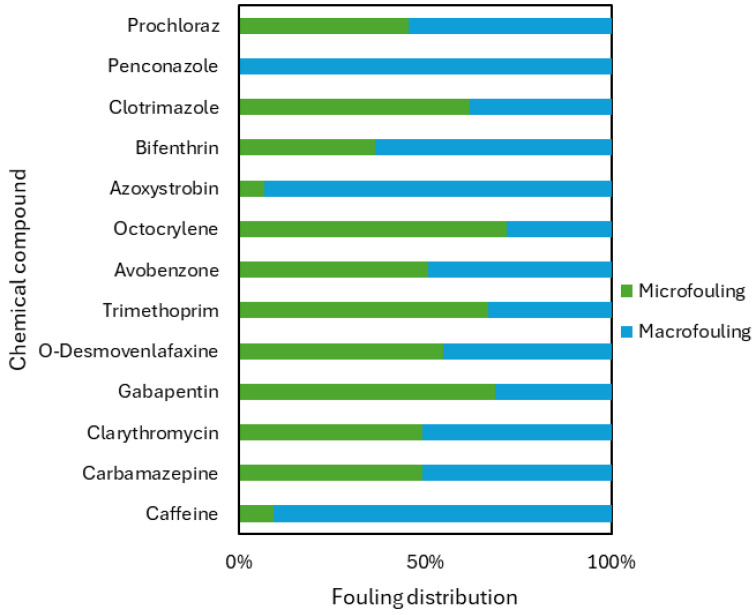
Profile of the relative distribution of individual CECs in the mesocosm macro- (blue) and microfouling (green).

When all the compounds are considered collectively, some of them, such as clarithromycin or prochloraz, display similar concentrations in both micro- and macrofouling. Conversely, compounds like caffeine, octocrylene, and azoxystrobin exhibit variations between fouling types. Figure 5 facilitates a comparative analysis of the detected compounds. Therefore, while the mesocosm proves useful as monitoring tool, the scale needs to be considered, whether micro or macro.

### 3.5. Passive Sampling and Sampling Comparison

Finally, passive sampling was performed to study the occurrence of CECs in the Malaga Mediterranean coastal area using HLB disks, as this is a more established and widely studied sampling method [5,6]. Passive sampling showed similar concentrations (Table S2) for certain compounds in relation to the biofilm mesocosm. The two sun agents presented the highest concentrations: 1.031 ± 0.111 ng/L for avobenzone and 0.27 ± 0.03 ng/L for octocrylene. These compounds were followed by caffeine, 0.090 ± 0.019 ng/L, other pharmaceuticals, and the pesticides, with the lowest concentration.

**Figure 6:**
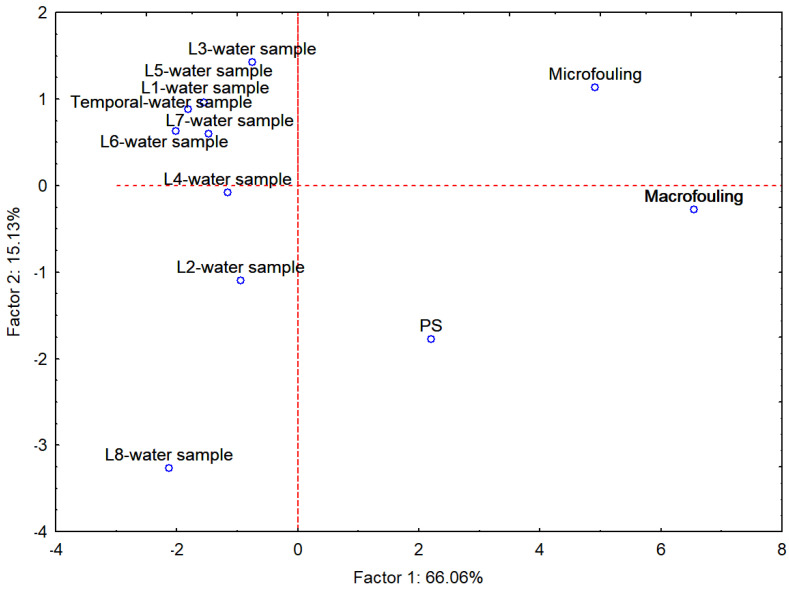
PCA of the sampling procedures (time-line grab sample, different location grab samples, mesocosm microfouling, mesocosm macrofouling and passive sampling (PS)) using thirteen CECs as variables.

Since only thirteen compounds were detected in both the mesocosm and passive samplers (PS), these were selected for the statistical analysis. For the time-line study, the mean concentration over the five sampling weeks was calculated. This mean value, together with the grab samples collected at each location, was considered as the water sampling data. Consequently, the analysis was limited to the following: time-line–water sampling (mean concentration across five consecutive weeks), location–water sampling (one per site), mesocosm–macrofouling, mesocosm–microfouling, and passive sampling. The thirteen compounds identified in the mesocosm biofilm were selected by an ANOVA, and a principal component analysis (PCA; Figure 6) was subsequently performed using these compounds as variables and the different sampling procedures as cases. The PCA scores are presented in Figure S2.

The two principal components together explain 81.19% of the variance in the dataset. Factor 1 clearly separates the water samples from the fouling samples, emphasizing the substantial differences between instantaneous water sampling and integrative approaches such as fouling. This axis is primarily driven by compounds like caffeine, octocrylene, or trimethoprim, reflecting their strong influence on water-phase profiles. Factor 2 further distinguishes mesocosm microfouling from both passive sampling and mesocosm macrofouling, indicating compositional divergence between early-stage biofilm communities and more complex fouling assemblages. Notably, macrofouling and passive sampling cluster closely, supporting the notion that passive samplers mimic bioaccumulation patterns in higher-trophic-level organisms, in line with Smedes et al. [33]. The separation of microfouling and macrofouling along Factor 2 also highlights distinct accumulation dynamics: early-colonizing biofilms versus mature assemblages such as mussels, barnacles, tunicates, and macroalgae, consistent with observed differences in compounds including caffeine and octocrylene. Furthermore, the intermediate position of the passive sampler-closer to macrofouling than to water samples demonstrates its effectiveness as an integrative monitoring tool that parallels organismal accumulation. This interpretation is reinforced by Martinaiou et al. [34], who showed that passive samplers, when used alongside spot sampling in marine aquaculture systems, successfully detected pharmaceuticals such as trimethoprim over a 10-month period, even when they remained undetected by water sampling.

In summary, this analysis underscores the superiority of fouling-based and passive sampling approaches for monitoring environmental exposure to contaminants. They provide a broader temporal integration and more ecologically relevant signatures of pollutant accumulation compared to point-in-time water sampling.

## 4. Conclusions

In conclusion, UV filters, particularly avobenzone and octocrylene, were detected at their highest concentrations, with elevated levels in beach areas compared to harbours. Pharmaceuticals followed in abundance, with caffeine, gabapentin, and o-desvenlafaxine being most prominent, likely reflecting remobilization processes and direct inputs from sewage and cruise activities. The detection of pesticides, especially bifenthrin, raises additional concerns regarding their extensive use and environmental persistence. Time-line analyses revealed no clear temporal trends for either pharmaceuticals or pesticides, suggesting a continuous influx of CECs into the marine environment, while also underlining the influence of sampling time. The mesocosm experiment, which assessed both micro- and macrofouling, showed overall similar contaminant profiles but significant differences for specific compounds, pointing to divergent accumulation dynamics among fouling communities. Passive sampling proved to be a valuable complementary tool for monitoring CECs in marine and transitional waters, exhibiting patterns comparable to mesocosm macrofouling. However, it should not be considered equivalent, as differences were observed between mesocosm microfouling and passive samplers, and deployment duration provides only a partial snapshot of contaminant occurrence.

Therefore, the use of biofilm mesocosms as sampling devices holds significant promise for advancing marine environmental monitoring, providing a more robust and integrative assessment of pollution impacts on coastal and marine ecosystems across diverse spatial and temporal scales.

## Figures and Tables

**Table 1:** Description of the measured CECs: chemical group, functional category, empirical formula, CAS number, highest *m*/*z* fragment, MS transition (quantifier and qualifier), and internal standard (with the deuterated (d) position) employed for each compound.

Chemical Compound	General Group	Functional Category	Empirical Formula	CAS No.	Highest *m*/*z* Fragment	MS Transition (Quantifier and Qualifier)	Internal Standard
Caffeine	Pharmaceuticals	Stimulant	C_8_H_10_N_4_O_2_	558-08-2	195.0	195.0–138.0 195.0–110.0	Caffeine-d10
Carbamazepine		Anticonvulsant	C_15_H_12_N_2_O	298-46-4	194.0	237.1–194.0 237.1–193.2	Carbamazepine-d10
Clarithromycin		Antibiotic	C_38_H_69_NO_13_	81103-11-9	748.5	748.5–590.4 748.5–158.1	Clarithromycin-d3
Erythromycin		Antibiotic	C_37_H_67_NO_13_	114-07-8	734.5	734.5–576.4 734.5–158.1	Erythromycin-d3
Gabapentin		Anticonvulsant	C_9_H_17_NO_2_	60142-96-3	172.1	172.1–154.1 172.1–137.0	Gabapentin-d4
O-Desvenlafaxine		Antidepressant	C_16_H_25_NO_2_	93413-62-8	163.8	264.2–107.0 264.2–77.0	O-Desvenlafaxine-d6
Ofloxacin		Antibiotic	C_18_H_20_FN_3_O_4_	82419-36-1	362.2	362.2–318.3 362.2–261.3	Ofloxacin-d3
Sulfamethoxazole		Antibiotic	C_10_H_11_N_3_O_3_S	723-46-6	156.0	254.0–156.0 254.0–108.4	Sulfamethoxazole-d13
Trimethoprim		Antibiotic	C_14_H_18_N_4_O_3_	738-70-5	291.2	291.2–258.0 291.2–230.1	Trimethoprim-d9
Venlafaxine		Antidepressant	C_17_H_27_NO_2_	99300-78-4	215.0	278.2–215.0 278.2–121.0	Venlafaxine-d6
Avobenzone	Sun agents	UV filter	C_20_H_22_O_3_	70356-09-1	161.0	311.0–161.0 311.0–135.0	Avobenzone-d3
Octocrylene		UV filter	C_24_H_27_NO_2_	6197-30-4	250.1	362.2–250.1 362.2–231.0	Octocrylene-d10
Oxybenzone		UV filter	C_14_H_12_O_3_	131-57-7	151.2	229.2–151.2 229.2–128.0	Oxybenzone-d5
Azoxystrobin	Pesticides	Fungicide	C_22_H_17_N_3_O_5_	131860-33-8	372.1	404.1–372.1 404.1–344.1	Azoxystrobin-d4
Clotrimazole		Fungicide	C_22_H_17_ClN_2_	23593-75-1	277.0	277.0–241.1 277.0–199.0	Clotrimazole-d10
Bifenthrin		Insecticide	C_23_H_22_ClF_3_O_2_	99267-18-2	181.1	442.1–181.1 440.1–198.2	Bifenthrin-d5
Fluconazole		Fungicide	C_13_H_12_F_2_N_6_O	86386-73-4	238.1	307.1–238.1 307.1–220.1	Fluconazole-d4
Miconazole		Fungicide	C_18_H_14_C_l4_N_2_O	22916-47-8	159.0	415.0–159.0 415.0–69.0	Miconazole-d5
Penconazole		Fungicide	C_13_H_15_Cl_2_N_3_	66246-88-6	159.0	284.1–159.0 284.1–70.0	Penconazole-d7
Prochloraz		Fungicide	C_15_H_16_Cl_3_N_3_O	67749-09-5	376.0	376.0–308.0 376.0–266.0	Prochloraz(ethylene)-d4

**Table 2:** Sampling points for the spatial analysis of Malaga coastal area and their main activities.

Sampling Code	Longitude	Latitude	Main Activity of the Area
L1	36.673905 N	4.449118 W	Beach/Swimming
L2	36.682097 N	4.444226 W	Beach/Swimming
L3	36.690012 N	4.440707 W	Beach/Swimming
L4	36.696688 N	4.436844 W	Beach/Swimming
L5	36.702469 N	4.426030 W	Swimming/Navigation
L6	36.701368 N	4.418906 W	Harbour/Navigation
L7	36.708868 N	4.418048 W	Harbour/Navigation
L8	36.717607	4.416588	Harbour/Navigation

**Table 3:** Validation of LC-MS/MS method for analysis of CECs.

Chemical Compound	r^2^	LOD (×10^−3^ ng/L)	LOQ (×10^−3^ ng/L)	R * (%)	RSD * (%)	R ** (%)	RSD ** (%)	ME (%)
Caffeine	0.9994	0.2	0.7	82.7	2.1	94.1	6.0	−35.5
Carbamazepine	0.9924	1.0	3.2	85.6	1.7	88.9	7.8	−43.5
Clarithromycin	0.9956	0.4	1.3	82.9	2.8	104.5	4.9	42.8
Erythromycin	0.9912	0.8	2.9	94.1	4.6	91.6	3.6	−39.3
Gabapentin	0.9969	1.1	3.3	109.8	5.4	85.1	8.5	30.1
O-Desvenlafaxine	0.9967	0.8	2.9	96.2	0.9	86.1	3.1	10.8
Ofloxacin	0.9914	0.1	0.6	91.4	7.9	97.9	1.1	22.4
Sulfamethoxazole	0.9966	0.6	2.0	112.5	5.1	103.7	6.3	−15.1
Trimethoprim	0.9923	0.2	0.7	84.9	3.2	111.9	3.9	−44.5
Venlafaxine	0.9986	0.3	1.0	110.8	2.5	88.5	6.4	−32.2
Avobenzone	0.9943	0.9	3.0	96.4	8.6	100.6	3.5	26.5
Octocrylene	0.9988	0.8	2.9	108.9	6.4	108.4	8.1	−18.7
Oxybenzone	0.9916	0.9	2.9	89.3	1.3	95.2	8.5	45.4
Azoxystrobin	0.9924	0.1	0.4	106.7	7.8	91.3	1.4	−30.5
Clotrimazole	0.9953	1.0	3.1	97.7	3.1	94.7	5.2	−31.3
Bifenthrin	0.9927	0.1	0.3	104.1	5.6	109.5	4.3	51.2
Fluconazole	0.9931	0.4	1.1	97.5	4.7	93.3	7.2	38.1
Miconazole	0.9945	0.6	2.0	111.6	5.0	107.8	4.9	26.1
Penconazole	0.9978	0.3	1.0	98.2	1.9	90.6	2.8	−16.1
Prochloraz	0.9980	0.1	0.2	113.4	2.8	103.4	1.6	27.5

Note: r^2^—correlation coefficient; LOD—limit of detection; LOQ—limit of quantification; R—recovery; RSD—relative standard deviation; *—spiking level 1 ng/L; **—spiking level 10 ng/L; ME—matrix effect.

**Table 4:** Pharmaceutical concentration ± standard deviation (SD) (ng/L) in the different sampling points.

Locations	L1	L2	L3	L4	L5	L6	L7	L8
Caffeine	0.313 ± 0.010	0.367 ± 0.012	0.376 ± 0.027	0.363 ± 0.016	0.391 ± 0.007	0.322 ± 0.014	0.358 ± 0.025	0.361 ± 0.019
Carbamazepine	0.185 ± 0.009	0.339 ± 0.031	0.191 ± 0.012	0.261 ± 0.010	0.134 ± 0.009	0.189 ± 0.014	0.246 ± 0.022	0.179 ± 0.008
Clarythromycin	0.309 ± 0.007	0.271 ± 0.022	0.270 ± 0.014	0.188 ± 0.016	0.237 ± 0.015	0.270 ± 0.020	0.342 ± 0.028	0.350 ± 0.017
Erythromycin	0.044 ± 0.008	0.050 ± 0.006	0.049 ± 0.007	0.050 ± 0.005	0.028 ± 0.004	0.052 ± 0.006	0.055 ± 0.002	0.032 ± 0.004
Gabapentin	0.298 ± 0.021	0.265 ± 0.014	0.283 ± 0.011	0.251 ± 0.015	0.314 ± 0.006	0.271 ± 0.012	0.292 ± 0.024	0.277 ± 0.026
O-Desmovenlafaxine	0.376 ± 0.019	0.198 ± 0.015	0.374 ± 0.016	0.364 ± 0.028	0.262 ± 0.018	0.334 ± 0.023	0.267 ± 0.029	0.235 ± 0.021
Ofloxacin	0.156 ± 0.011	0.201 ± 0.005	0.286 ± 0.017	0.131 ± 0.021	0.348 ± 0.022	0.314 ± 0.016	0.285 ± 0.009	0.222 ± 0.030
Sulfamethoxazole	0.246 ± 0.013	0.361 ± 0.026	0.293 ± 0.012	0.360 ± 0.034	0.243 ± 0.017	0.211 ± 0.019	0.362 ± 0.020	0.284 ± 0.016
Trimethoprim	0.073 ± 0.008	0.099 ± 0.006	0.096 ± 0.009	0.084 ± 0.004	0.081 ± 0.006	0.112 ± 0.005	0.062 ± 0.04	0.120 ± 0.009
Venlafaxine	0.128 ± 0.004	0.200 ± 0.032	0.321 ± 0.023	0.365 ± 0.026	0.340 ± 0.029	0.142 ± 0.019	0.270 ± 0.030	0.174 ± 0.011

**Table 5:** Pesticide concentration ± SD (ng/L) in the different sampling points.

Locations	Azoxystrobin	Bifenthrin	Clotrimazole	Fluconazole	Miconazole	Penconazole	Prochloraz
L1	<LOQ	0.008 ± 0.001	<LOQ	<LOQ	<LOQ	<LOQ	0.002 ± 0.001
L2	<LOQ	0.023 ± 0.004	0.003 ± 0.001	<LOQ	<LOQ	<LOQ	0.002 ± 0.001
L3	<LOQ	0.007 ± 0.001	<LOQ	<LOQ	<LOQ	<LOQ	0.003 ± 0.001
L4	<LOQ	0.008 ± 0.001	0.003 ± 0.001	<LOQ	0.002 ± 0.001	0.003 ± 0.001	0.003 ± 0.001
L5	<LOQ	0.027 ± 0.003	<LOQ	0.002 ± 0.001	0.002 ± 0.001	<LOQ	0.004 ± 0.001
L6	<LOQ	0.010 ± 0.001	<LOQ	0.002 ± 0.001	0.004 ± 0.001	<LOQ	0.002 ± 0.001
L7	<LOQ	0.015 ± 0.002	<LOQ	<LOQ	<LOQ	<LOQ	0.003 ± 0.001
L8	<LOQ	0.014 ± 0.001	<LOQ	0.003 ± 0.001	<LOQ	0.004 ± 0.001	0.002 ± 0.001

Note: <LOQ means below the limit of quantification (LOQ); thus, those compounds were not quantified.

**Table 6:** Risk assessment of CECs during five consecutive weeks.

Compound	Week 1	Week 2	Week 3	Week 4	Week 5
Caffeine	L.R.	L.R.	L.R.	L.R.	L.R.
Carbamazepine	N.R.	N.R.	N.R.	N.R.	N.R.
Erythromycin	N.R.	N.R.	N.R.	N.R.	N.R.
Gabapentin	N.R.	N.R.	N.R.	N.R.	N.R.
O-desvenlafaxine	L.R.	L.R.	L.R.	L.R.	L.R.
Sulfamethoxazole	N.R.	N.R.	N.R.	N.R.	N.R.
Trimethoprim	N.R.	N.R.	N.R.	N.R.	N.R.
Venlafaxine	L.R.	L.R.	L.R.	L.R.	N.R.
Avobenzone	H.R.	H.R.	H.R.	H.R.	H.R.
Octocrylene	N.R.	N.R.	N.R.	N.R.	N.R.
Oxybenzone	N.R.	N.R.	N.R.	N.R.	N.R.
Azoxystrobin	N.R.	L.R.	L.R.	L.R.	N.R.
Bifenthrin	H.R.	H.R.	H.R.	H.R.	H.R.
Clotrimazole	N.R.	N.R.	N.R.	N.R.	N.R.
Fluconazole	N.R.	N.R.	N.R.	N.R.	N.R.
Miconazole	N.R.	N.R.	N.R.	N.R.	N.R.
Penconazole	N.R.	N.R.	N.R.	N.R.	N.R.
Prochloraz	N.R.	N.R.	N.R.	N.R.	N.R.

Note: four risk levels were considered: RQ < 0.01—no risk (N.R.; green color); 0.01 < RQ < 0.1—low risk (L.W.; orange color); 0.1 < RQ < 1—moderate risk (M.R.); RQ > 1—high risk (H.R.; red color).

## Data Availability

The original contributions presented in this study are included in the article/Supplementary Material. Further inquiries can be directed to the corresponding author(s).

## Supplementary Materials

The following supporting information can be downloaded at: https://www.mdpi.com/article/10.3390/jox15050149/s1. Figure S1: Schematic overview of the experimental setup. Figure S2: Score of the PCA using 13 of the measured CECs. Table S1: Concentration ± SD (ng/L) of the pharmaceuticals and pesticides in the L5 sampling site during five consecutive weeks. Table S2: Concentration ± SD (ng/L) of the compounds detected by passive sampling.

## Abbreviations

The following abbreviations are used in this manuscript:

CECs	Contaminants of emerging concern
PS	Passive sampling
SPE	Solid-phase extraction
HPLC-MS	High-performance liquid chromatography couple with triple quadrupole mass spectrum
UVF	Ultraviolet filter
LOD	Limit of detection
LOQ	Limit of quantification
ME	Matrix effect
RSD	Relative standard deviation
r^2^	Correlation coefficient
N.R.	No risk
L.R.	Low risk
M.R.	Moderate risk
H.R.	High risk
