# Supplementary material for: Multisampling Strategies for Determining Contaminants of Emerging Concern (CECs) in the Marine Environment
Source: J Xenobiot. 2025 Sep 15;15(5):149. doi: 10.3390/jox15050149 (PMC12452650; doi:10.3390/jox15050149)
Supplement: Supplementary file 1 [file jox-15-00149-s001.zip › jox-3842272-supplementary.pdf]

Supplementary Materials: Multisampling Strategies for Determining Contaminants of Emerging Concern (CECs) in the Marine Environment

Enrique J. Díaz-Montaña and Sofía Domínguez-Gil

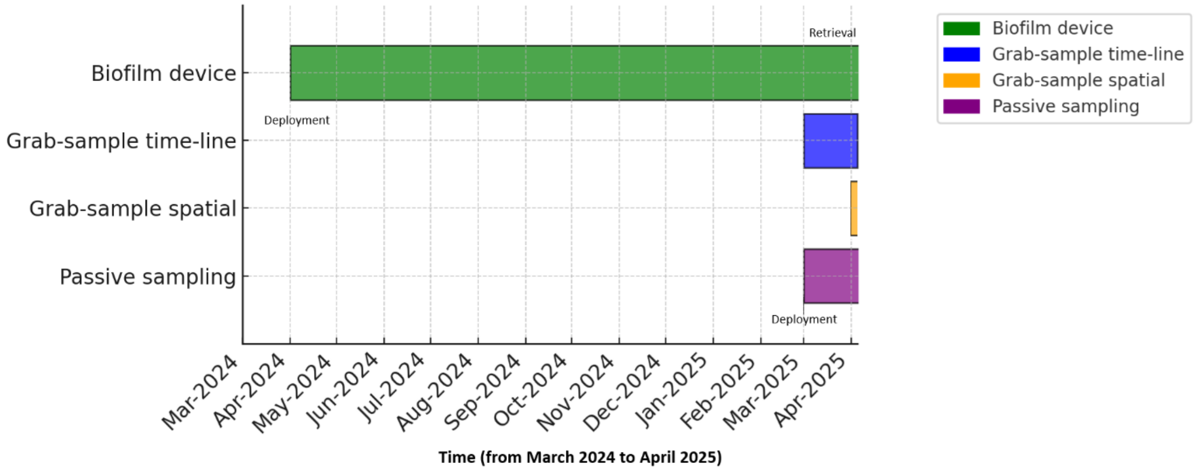

Figure S1. Schematic overview of the experimental setup.

Table S1. Concentration ± SD (ng/L) of the pharmaceuticals and pesticides in the L5 sampling site during five consecutive weeks.

Compound	Week 1	Week 2	Week 3	Week 4	Week 5
Caffeine	0.271 ± 0.020	0.093 ± 0.007	0.163 ± 0.009	0.158 ± 0.021	0.203 ± 0.010
Carbamazepine	0.006 ± 0.001	0.006 ± 0.001	0.012 ± 0.004	0.009 ± 0.001	0.004 ± 0.001
Clarythromycin	0.007 ± 0.001	0.007 ± 0.001	0.010 ± 0.001	0.007 ± 0.001	0.003 ± 0.001
Erythromycin	0.002 ± 0.001	0.002 ± 0.001	0.002 ± 0.001	0.001 ± 0.001	0.001 ± 0.001
Gabapentin	0.040 ± 0.004	0.041 ± 0.006	0.042 ± 0.008	0.038 ± 0.002	0.043 ± 0.001
O-Desmovenlafaxine	0.040 ± 0.005	0.042 ± 0.005	0.056 ± 0.005	0.042 ± 0.002	0.018 ± 0.001
Ofloxacin	0.001 ± 0.001	< LOQ	< LOQ	< LOQ	< LOQ
Sulfamethoxazole	0.004 ± 0.001	0.004 ± 0.001	0.005 ± 0.001	0.005 ± 0.001	0.002 ± 0.001
Trimethoprim	0.001 ± 0.001	0.001 ± 0.001	0.002 ± 0.001	0.001 ± 0.001	0.001 ± 0.001
Venlafaxine	0.014 ± 0.002	0.016 ± 0.001	0.027 ± 0.003	0.016 ± 0.001	0.005 ± 0.001
Avobenzone	0.280 ± 0.011	0.361 ± 0.016	0.222 ± 0.014	0.236 ± 0.011	0.284 ± 0.026
Octocrylene	0.100 ± 0.018	0.100 ± 0.011	0.100 ± 0.008	0.100 ± 0.009	0.100 ± 0.012
Oxybenzon	0.015 ± 0.003	< LOQ	< LOQ	< LOQ	< LOQ
Azoxystrobin	0.001 ± 0.001	0.002 ± 0.001	0.002 ± 0.001	0.001 ± 0.001	0.001 ± 0.001
Bifenthrin	0.065 ± 0.006	0.079 ± 0.004	0.074 ± 0.009	0.030 ± 0.005	0.077 ± 0.004
Clotrimazole	< LOQ	< LOQ	< LOQ	< LOQ	< LOQ
Fluconazole	0.004 ± 0.001	0.004 ± 0.001	0.005 ± 0.001	0.004 ± 0.001	0.001 ± 0.001
Miconazole	< LOQ	< LOQ	< LOQ	< LOQ	< LOQ
Penconazole	< LOQ	< LOQ	< LOQ	< LOQ	< LOQ
Prochloraz	< LOQ	< LOQ	< LOQ	< LOQ	< LOQ

Note: < LOQ means that the signal is below the limit of quantification, thus those compounds where not able to be quantified.

Table S2. Concentration ± SD (ng/L) of the compounds detected by passive sampling.

Chemical compound	Concentration ± SD (ng/L)
Caffeine	0.087 ± 0.011
Carbamazepine	< LOQ
Clarythromycin	0.004 ± 0.001
Gabapentin	0.038 ± 0.008
O-Desmovenlafaxine	< LOQ
Trimethoprim	0.002 ± 0.001
Avobenzone	1.030 ± 0.061
Octocrylene	0.267 ± 0.044
Azoxystrobin	0.003 ± 0.001
Bifenthrin	0.001 ± 0.001
Clotrimazole	0.001 ± 0.001
Penconazole	< LOQ
Prochloraz	< LOQ

Note: < LOQ means that the signal is below the limit of quantification, thus those compounds where not able to be quantified.

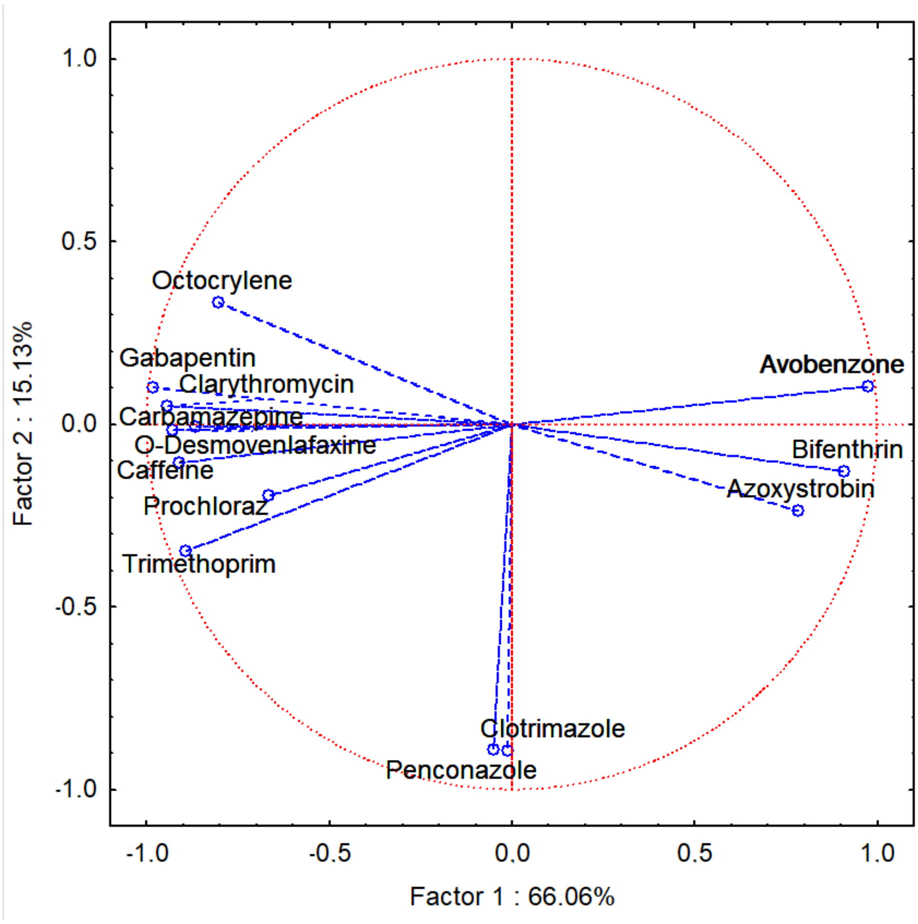

Figure S2. Score of the PCA using 13 of the measured CECs.
